# Laparoscopy: A Better Approach for Perforated Duodenal Ulcer

**DOI:** 10.7759/cureus.10953

**Published:** 2020-10-15

**Authors:** Muhammad Faisal Murad, Rafeya Khan, Maham Tariq, Ayesha Akram, Ronald C Merrell, Asif Zafar

**Affiliations:** 1 Surgery, Maroof International Hospital, Islamabad, PAK; 2 Surgery, Holy Family Hospital, Rawalpindi, PAK; 3 Internal Medicine, Rawalpindi Medical University, Rawalpindi, PAK; 4 Surgery, Virginia Commonwealth University, Richmond, USA; 5 Surgery, Al Nafees Medical College and Hospital, Islamabad, PAK

**Keywords:** laparoscopy, graham patch repair, perforated duodenal ulcer

## Abstract

Background

Laparoscopic surgery is becoming the gold standard for most abdominal surgeries in recent times. Laparoscopic repair of perforated duodenal ulcer (PDU), however, is still an area of debate. The purpose of this study was to evaluate the safety and efficacy of laparoscopic repair of PDU versus open repair.

Methods

In this cross-sectional study, patients were consecutively sampled. Out of 101 patients with clinically diagnosed PDU, 36 patients underwent laparoscopic Graham patch repair and 65 underwent open Graham patch repair in a tertiary care academic hospital. Open repair was via upper midline incision, and laparoscopic repair by the three-port technique. The following stages were calculated: operative time, duration of postoperative analgesia, time taken to mobilize, and patient length of stay after the operation.

Results

The mean operative time was somewhat longer in the laparoscopy group compared to the open repair group (74.01 vs 56.17 minutes, respectively). Mean postoperative analgesia requirement, time taken to mobilize, and hospital stay were significantly shorter after laparoscopy than after open repair (1.21 days, 9.32 hours, and 3.12 days vs 3.83 days, 16.20 hours, and 4.85 days, respectively). Three patients (8%) in the laparoscopy group and 35 (54%) in the open repair group had postoperative complications.

Conclusions

Laparoscopic repair of PDU is a safe approach and better than open repair in terms of operative time with the right level of expertise only, postoperative analgesia requirement, mobilization, duration of hospital stay, and incidence of postoperative respiratory and wound complications.

## Introduction

Since the past decade, there has been an extensive change in the approach to abdominal surgeries from laparotomy to minimal access surgery. Laparoscopy has now become the gold standard in most of the elective abdominal procedures [1,2]. However, there is still a debate on the use of laparoscopic approach in emergency scenarios such as peritonitis. On the one hand, previously published studies warn about pneumoperitoneum-induced bacteremia and delayed recovery [3,4], whereas, on the other hand, there are advantages of early mobilization, less postoperative analgesia requirement, and shorter hospital stay [5-7]. One such condition where laparoscopic approach can be considered an option is perforated duodenal ulcer (PDU).

With the success of medical therapy the incidence of emergency presentations and morbidity associated with duodenal ulcer disease have reduced dramatically, and elective duodenal ulcer surgery has virtually been abandoned [8]. But PDU is one complication of the disease that is still not an uncommon presentation in the emergency department (ED). The incidence of this disease is about 1 in 600 emergency presentations during usual months, but various studies published in Pakistan have observed that the incidence of PDU becomes higher during the holy month of Ramadan. Apart from the various general factors such as use of non-steroidal anti-inflammatory drugs, smoking, stress-related disorders, and Helicobacter pylori infection contributing most of the cases [9-12], certain other factors such as prolonged fasting contribute to its higher incidence in Ramadan [13].

Laparoscopy was introduced in the ED of Holy Family Hospital, Rawalpindi, Pakistan, three years back. It has been used extensively to perform laparoscopic appendectomies during this time. In all young females presenting with pain right iliac fossa, laparoscopic exploration was the approach in our ED. Subsequently, laparoscopy has been initiated for patients presenting with peritonitis. It is being used as a tool in the management of patients with peritonitis secondary to perforated appendix, PDU, and enteric perforation. This study highlights the experience of managing patients with peritonitis secondary to PDU laparoscopically during the last two years at Surgical Unit II, Holy Family Hospital.

## Materials and methods

In Surgical Unit II, Holy Family Hospital, three teams, F, A, and Q, are working under the supervision of one senior registrar each. Team F works via laparoscopic approach on Tuesdays, while Teams A and Q perform the open procedures on Thursdays and Saturdays, respectively, for PDU. We conducted a cross-sectional study from June 2017 to July 2019 on patients presenting to the ED with a clinical diagnosis of peritonitis secondary to PDU (sudden-onset abdominal pain, abdominal 'board-like' rigidity, tenderness or guarding on physical examination). The study was approved by the Institutional Review Board of Rawalpindi Medical University. Patients with previous midline laparotomy wounds, those with the presence of major comorbidities (American Society of Anesthesiologists [ASA] III-IV), patients in shock, and those with type 2 respiratory failure were excluded. Based on this criteria, 101 patients aged 16-60 years were then included. By consecutive sampling, 36 patients were assigned to the laparoscopic group and 65 patients to the open group for Graham patch repair, and subsequently operated.

Laparotomy was performed with midline incision. After initial exploration and confirmation of diagnosis, fluid was drained and peritoneal lavage was performed. Omental patch was selected and plugged to the perforation via full thickness suture. In laparoscopic technique, a three-port technique was used in almost all cases. A 10-mm optical trocar was introduced through the umbilicus using the open technique. Two 5-mm working trocars were introduced in the midclavicular line on both sides almost at the level of the umbilicus. The procedure started with an initial exploration of the four abdominal quadrants followed by confirmation of diagnosis by localization of the perforation. The fluid was drained followed by peritoneal lavage with normal saline. The omentum was then grasped and its free edge was brought to the perforation where it was anchored with one stitch. Two additional Vicryl 2/0 seromuscular longitudinal stitches were placed to plug the omentum. A 16-Fr drain was placed routinely in right subhepatic space in both the laparoscopic and open groups.

All patients were given IV diclofenac sodium thrice a day for 48 hours as routine postoperative analgesia. After 48 hours, if required, the analgesia duration was extended on the patient’s demand. If during this time, any patient required analgesia in addition to routine diclofenac sodium, Nalbin injection was given and documented as rescue analgesia. Patients were attempted to mobilize 6 hours after surgery. In case of difficulty, the same was repeated every 6 hours. The discharge criteria included tolerating liquid diet and pain-free mobilization.

The process that took place in the operating rooms was divided into five stages to collect the respective variables: (1) operative time; (2) time taken to mobilize the patient after the operation; (3) time taken by the patient to resume normal activities after the operation; (4) duration of postoperative analgesia; and (5) time between the end of the procedure and patient exit from the hospital. The collected data were submitted to a descriptive analysis. Statistical analysis was performed using the IBM Statistical Package for Social Sciences (SPSS), Version 23.0 (IBM Corp., Armonk, NY, USA). Postoperative respiratory and wound complications were also taken into account, and percentages were calculated.

## Results

A total of 101 patients with PDU were treated by Graham patch repair technique during the study time. Of these, 36 were treated laparoscopically and 65 with laparotomy. The mean age of the patients was 36.3 ± 5.7 years, and the male-to-female gender distribution ratio was 6:1. The mean duration of peritonitis at the time of presentation was 28.6 ± 9.7 hours. There were statistical differences in the following five variables (p < 0.001). The mean operative time for laparoscopy and laparotomy was 74.01 ± 4.12 minutes and 56.17 ± 6.33 minutes, respectively. Mean postoperative analgesia requirement was 1.21 ± 0.04 days and 3.83 ± 1.35 days for laparoscopy and laparotomy, respectively. Mobilization of the patient was achieved within a mean of 9.32 ± 2.78 hours in laparoscopic group and 16.20 ± 3.56 hours in open group. Mean for the return to normal activity was 2.34 ± 0.75 days for the laparoscopy group and 3.94 ± 1.15 days for the open group. Duration of postoperative hospital stay was 3.12 ± 0.56 days and 4.85 ± 1.65 days for laparoscopic and open groups, respectively. Table 1 summarizes these results.

**Table 1:** Laparoscopy versus laparotomy for perforated duodenal ulcer *Difference is significant at 5% level of significance (after applying independent Student’s t-test).

Variable	Laparoscopic group (n=36)	Laparotomy group (n=65)	p-Value*
Mean operative time (minutes)	74.01 ± 4.12	56.17 ± 6.33	0.000
Mean postoperative analgesia requirement (days)	1.21 ± 0.04	3.83 ± 1.35	0.000
Mean time taken for mobilization of the patient (hours)	9.32 ± 2.78	16.2 ± 3.56	0.000
Mean time taken for return to normal activity (days)	2.34 ± 0.75	3.94 ± 1.15	0.000
Mean postoperative hospital stay (days)	3.12 ± 0.56	4.85 ± 1.65	0.000

As shown in Table 2, two patients (6%) in the laparoscopic group and 20 patients (31%) in the open repair group suffered postoperative respiratory complications, whereas wound infections were confirmed in only one patient (3%) following laparoscopic repair and in 15 patients (23%) following open repair.

**Table 2:** Comparing postoperative complications in laparoscopic and open repair for perforated duodenal ulcer

Comparison of	Laparoscopic group (n=36)	Open group (n=65)
Respiratory complications	2 (6%)	20 (31%)
Wound complications	1 (3%)	15 (23%)
Total	3 (8%)	35 (54%)

Conversion from laparoscopic to open was performed in one case. The reason for conversion was inability to localize the perforation. The perforation was sealed.

## Discussion

The debate of laparoscopic approach compared to laparotomy in PDU even after a decade is yet to be concluded. There are studies in which laparoscopy has strongly been recommended for PDU repair [5,6], whereas others conclude that laparoscopic approach has little or no benefits [4]. There are general concerns regarding laparoscopic approach, which includes working in a difficult two-dimensional environment with loss of depth perception and haptic sensations along with restricted range of movements. Numerous published studies have proven that the time spent and skill acquired in the training labs help surgeons overcome these general laparoscopic concerns in operating rooms [14,15].

Among the specific concerns regarding laparoscopic approach to PDU, the first concern highlighted was of pneumoperitoneum-induced bacteremia [16]. Laparoscopy in peritonitis may be related to an increased risk of bacteremia due to theoretical knowledge of translocation of bacteria into the systemic circulation under the increased pressure of carbon dioxide in the peritoneum [3]. However, Robertson's study denies this relationship by documenting no such incidence [4]. Our study also offers the reassurance of no such occurrence in any of the patients treated laparoscopically. The second concern pointed out in the initial studies was prolonged duration of laparoscopic procedure as compared to open laparotomy [17,18]. Longer duration of procedure in these critical patients means more chances of complications. Most of the general factors mentioned previously contribute towards longer duration of the procedure. However, a randomized controlled trial by Siu et al. showed that laparoscopy actually takes less time than open repair in expert hands [6]. In our experience, the regular use of laparoscopic approach gradually reduced the operative time and subsequently it came close to open procedure. Studies by Bertleff and Lange and by Lunevicius and Morkevicius highlighted another concern that the incidence of leakage at the repair site is more prevalent in laparoscopic omental patch repair despite strict exclusion criteria [5,19]. Leakage from the operation site ultimately means more readmissions and re-explorations after laparoscopy. In our experience, no such incidence of leakage after the laparoscopic Graham patch repair was seen. On the other hand, decreased pain and less analgesia requirement are the most agreed upon factors for the edge of laparoscopy over open in the majority of studies [20]. Siu et al. document lesser postoperative pain in laparoscopically treated PDU patients on the basis of the visual analog scale [6]. Robertson et al. too agree on the lesser opiate use and requirement in laparoscopically treated patients [4]. Our study also holds the same results, with less postoperative analgesia requirement measured on the scale set in the protocol for pain management. Matsuda et al. report decreased postoperative pain, rapid recovery, and fewer and less severe complications in laparoscopically treated patients of PDU [7]. Our study also shows that just one patient (3%) treated laparoscopically presented with any late wound complication compared to laparotomy where the comparative incidence was significantly higher (23%). Another common postoperative complication is respiratory. Our study had two patients (6%) in the laparoscopic group presenting with postoperative respiratory complications, this being significantly low in comparison to 20 patients (31%) in the laparotomy group presenting with postoperative respiratory complications. Bertleff and Lange’s study favors early mobilization and return to normal activity as significant factors achieved in patients who underwent laparoscopy [5]. We achieved an earlier mobilization and return to normal activity in laparoscopically treated patients compared to laparotomy during the course of our study too.

The European Association for Endoscopic Surgery in their 2006 recommendation advocates diagnostic laparoscopy and laparoscopic repair for cases where symptoms and diagnostic findings are suggestive of perforated peptic ulcer and the patient is fit to undergo laparoscopy [21].

## Conclusions

With the recent advancements in the field of minimally invasive surgery with good resolution laparoscopes, laparoscopic surgery simulation, and training outside the operating room, laparoscopy is yielding more and more promising results such as less operative time, less postoperative analgesia requirement, earlier mobilization, shorter duration of hospital stay, and lower incidence of postoperative respiratory and wound complications, and it should be considered for patients with PDU provided that the patient is fit to undergo laparoscopy and necessary expertise are available at the center. We recommend the implementation of laparoscopy over laparotomy for PDU.
